# Split hypoglossal facial anastomosis for facial nerve palsy due to skull base fractures: A case report

**DOI:** 10.1016/j.amsu.2020.08.056

**Published:** 2020-09-09

**Authors:** Eko Prasetyo, Maximillian Christian Oley, Muhammad Faruk

**Affiliations:** a Division of Neurosurgery, Department of Surgery, Faculty of Medicine, University Sam Ratulangi, Manado, Indonesia; b Division of Neurosurgery, Department of Surgery, R. D. Kandou Hospital, Manado, Indonesia; c Department of Surgery, Faculty of Medicine, Hasanuddin University, Makassar, Indonesia

**Keywords:** Hypoglossal nerve, Facial nerve, Facial palsy, Traumatic brain injury, Case report

## Abstract

**Introduction:**

Traumatic brain injury (TBI) is the most prevalent causes of morbidity and mortality worldwide. The biomechanics of primary TBI involve a direct impact, practically extended to the base of the skull, and most of the skull base fractures (SBF) are identified in anterior and medial cranial fossa. Furthermore, those predicted in the medial area are related to fissures from temporal bones.

**Presentation of case:**

We report two cases of right facial nerve palsy initiated by SBF's, which were diagnosed and treated at our institution. The 3D CT evaluation in our first case showed a longitudinal fracture of the right petrosal bone, which was longitudinal and transverse for the second case. Two cases of facial nerve palsy were managed with split hypoglossal facial anastomosis to restore functional reanimation. All patients were adequately achieved after the procedure, and the hypoglossal nerve function was preserved.

**Conclusion:**

Split hypoglossal facial anastomosis technique was used to treat patients with facial nerve paralysis resulting from SBF's. This was to achieve good recovery outcome, in terms of facial reanimation and preservation of tongue function.

## Highlights

• A skull base fracture (SBF) is about 4% of all cases Traumatic brain injury (TBI).
• SBF which frequently occurs in the petrous part of the temporal bone, is implicated in facial nerve palsy.
• Split hypoglossal facial anastomosis technique showed good recovery of facial reanimation with HB scale assessment.

## 1 Introduction

Traumatic brain injury (TBI) is an important contributor to morbidity and mortality. This condition has recently been ascribed as one of the most prominent medical problems in society [1,2]. Furthermore, about 4% of all cases include skull base fractures (SBF) [3], which frequently occurs in the petrous part of the temporal bone, after high-energy impact trauma [4]. However, the manifestation at the middle area of SBF's (7–10%) is implicated in facial nerve damage, as these route through temporal bone [5,6]. The electrophysiologic testing and 3 D CT have been identified as valuable tools in the evaluation of anatomy, pathomechanism, prognosis and surgical planning [7]. Moreover, split hypoglossal facial anastomosis is a potent and dependable technique adopted in surgical facial animation [8]. This case has been reported in line with the SCARE 2018 guidelines [9].

## 2 Case presentation

**Fig. 1:**
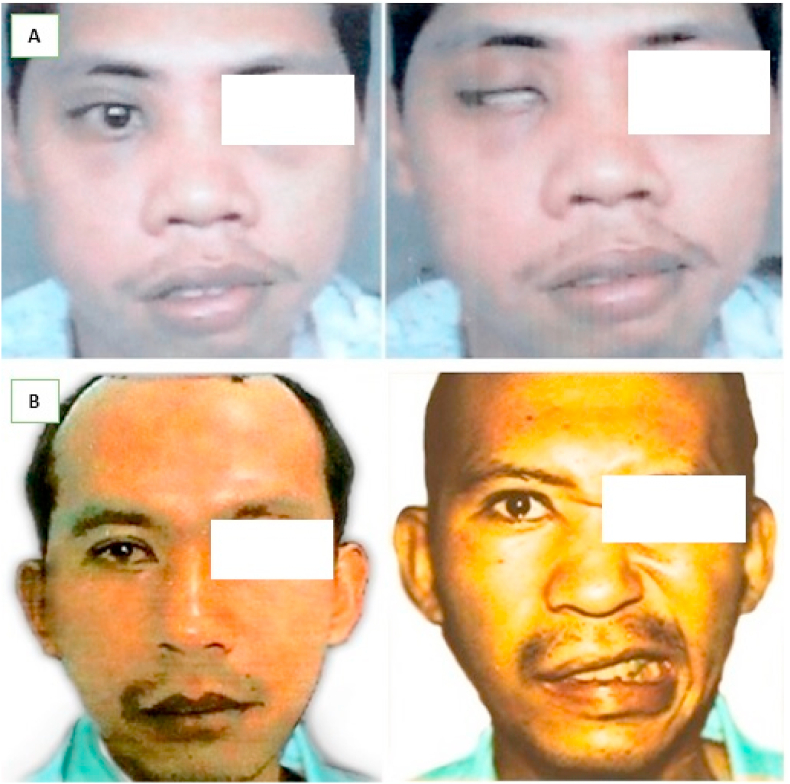
The condition of preoperative patients was examined using HB scale assessment, with HB III scale result for the first patient (A) and HB IV scale result for the second patient (B).

**Fig. 2:**
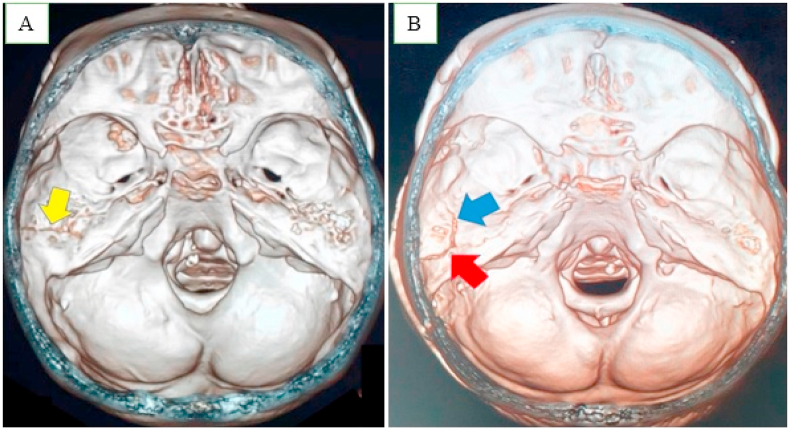
The 3D CT scan: A). a longitudinal fracture at first case (yellow arrow) and B). a longitudinal (red arrow) and transverse (blue arrow) fracture of petrous bone. (For interpretation of the references to colour in this figure legend, the reader is referred to the Web version of this article.)

This report presents 2 male patients 28 (Fig. 1A) and 30 (Fig. 1B) years old with right facial nerve palsy initiated by SBF's. The 3D CT evaluation in patient no.1 showed a longitudinal fracture of the right petrosal bone (Fig. 2A), which was longitudinal and transverse (Fig. 2B) for a second patient. Furthermore, the paralysis ensued immediately after trauma and is measured using the House Brackman scale as (HB)-III and HB-IV, respectively. Based on electrophysiological examination, degeneration and facial electromyography (EMG) provided fibrillation potentials on the 2nd week. Subsequently, similar results were obtained after 6 months of rehabilitation, electrophysiological examination and clinical re-evaluation (HB scale).

**Fig. 3:**
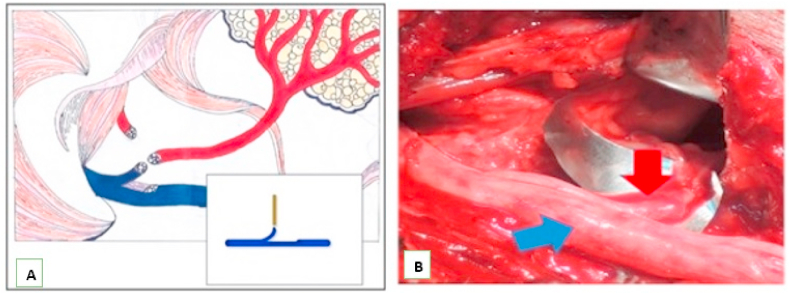
Scheme of split longitudinal hypoglossal nerve (blue) anastomosis to facial nerve (red). (For interpretation of the references to colour in this figure legend, the reader is referred to the Web version of this article.)

Therefore, we decided to perform the split hypoglossal facial anastomosis technique on both patients for the purpose of facial reanimation (Fig. 3A). The surgical microscopic magnification shows the presentation of hypoglossal nerve, characterized by longitudinal dissected retrograde (arrow, blue), spliced to connect with the proximal facial nerve (arrow, red), alongside several epineural sutures without tension and indications of thrombin fibrine glue (Fig. 3B). Furthermore, antibiotics were administered post operatively, and both patients were discharged in good recovery on the 10th day.

**Fig. 4:**
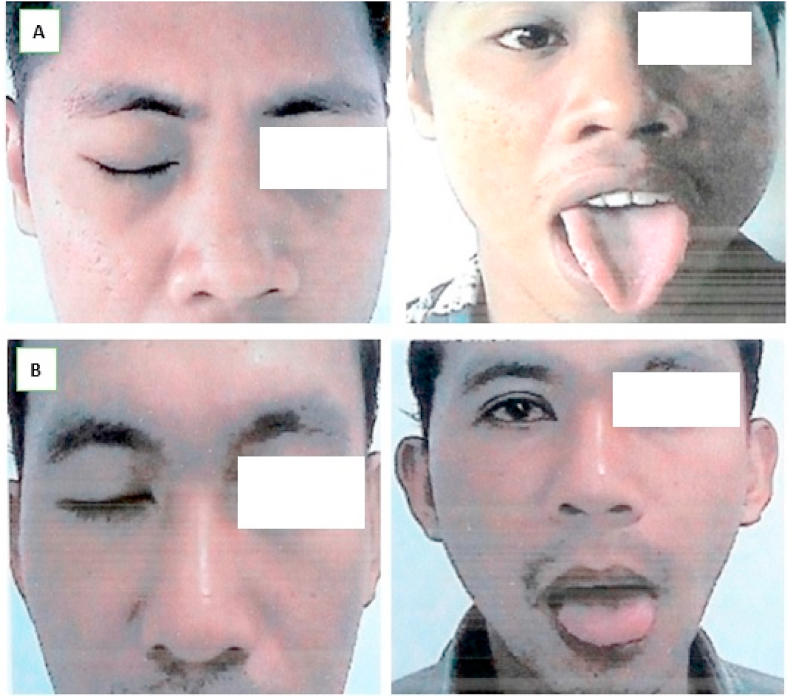
The condition of the postoperative patient was examined using HB scale assessment, the first patient with HB I scale results (A) and the second patient with HB I scale results (B).

Evaluation was conducted 6 months after surgery, using electrophysiological examination and HB scale assessment. The result showed a satisfactory clinical assessment with HB-II scale, alongside the absence of tongue dysfunction. Furthermore, proper rehabilitation was ascertained on the 10 months, evidenced by a HB-I scale (Fig. 4A and B).

## 3 Discussion

The calvarium comprises the vault, as well as the skull base, encompassing the paired orbital plates, with the cribriform in the middle. This also contains the sphenoid bone with lesser anterior and greater posterior wings, alongside parts of the squamous temporal, petrous, and occipital bones posteriorly [10]. A skull base fracture (SBF) is defined as any fracture involving the floor of the anterior, middle and posterior cranial fossa, assumed to originate from either a direct local or remote force impact [8]. This has been implicated in approximately 4% of TBIs [3], and also accounts for about 19% of all skull fractures [11]. In addition, blunt trauma is accountable for over 90% of all SBF's [12], with most injuries located in the anterior fossa (51%) [13]. Faried et al., reported on the occurrence of around 69.4% of cases in the anterior fossa, while 27.5% was in the middle cranial fossa [14]. Mokolane et al. described the center area as the most common target point, and is assumed as the weakest area, due to the presence of neurovascular foramina and thin bones [13,15]. However, the most frequently fractured spots, as observed in 14–22% of all cases were in the temporal bone or laterobasal fractures, resulting from high energy blunt impact [16]. This is congruent with other studies, where 30–47% was documented [13].

The temporal bone is formed in five portions, including the squama as the lateral wall, alongside the mastoid, petrous, tympanic and styloid [4]. In addition, possible fractures are classified according to the relationship with the long axis of the petrous pyramid, comprising the longitudinal, transverse and mixed. Approximately 80–90% of temporal bone fractures are longitudinal, with the crack line assumed to run along the roof of the external auditory canal, while 10–20% are transverse to the long axis of the petrous temporal bone [4,17]. Moreover, the facial nerve maintains a potentially precarious route through the temporal bone. Hence, there is a high liability to injury after the experience of skull fracture at this level [18]. The longitudinal forms have been associated with facial nerve palsy, where 20–30% of cases result from lateral basal injury to nerves located within the facial canal, commonly in the geniculate ganglion region [17,19]. Conversely, transverse temporal bone fractures are much rarer, and majorly occur after severe trauma to the occiput, and approximately 50% of patients manifest immediate facial nerve paralysis [19].

The cases evaluated in this study showed longitudinal and transverse fractures of the petrosus after 3D CT examination. This assessment tool is useful in the detection of fractures as well as any facial deformity, and also assists in reconstruction surgical planning [7]. The classification of petrous bone fractures relies on clinical finding and confirmation by CT [17]. However, pathologic entities located in and around the skull base are easily overlooked on conventional CT images obtained at the Emergency Department [10]. The 3 D version a gold standard, as the images rendered are significantly improved, hence the application is suitable in the radiological diagnosis of SBF's [3,17,20].

Various scoring system have been devised for grading the severity of the facial nerve impairment. The function was evaluated pre and post operatively, using the grading system of House and Brackman (HB) scale. This is assumed to be the most universally accepted measure, due to the simplicity, reproducibility and low inter-observer variability [21]. The Facial Nerve Disorders Committee recommended the application of this system during the evaluation and report of all recoveries [22]. Furthermore, gross facial appearance was assessed at rest, with movement, during forehead gestures, and motion around the eyes and mouth. The results show a gradation of facial nerve function from HB I to VI on the scale [[22], [23], [24]].

Pre operatively, patient no.1 (HB-III scale, moderate dysfunction) was better than no.2 (HB-IV scale, moderately severe dysfunction). This is possibly explained by the individuals’ state, comprising the combination longitudinal and transverse temporal bone fractures, indicating a high energy blunt impact.

Also, it is important to regularly perform clinical assessment and electrophysiological testing in the form of electroneurography (ENOG) and electromyography (EMG), to determine the timing onset of facial weakness and whether the injury is partial or complete [13]. However, surgery is usually recommended on instances where no regeneration potentials are noted with EMG, and after a prognosis of total paralysis with over 90% reduction in amplitude of motor response, as confirmed by ENOG [5,25]. Furthermore, an electrophysiological examination conducted on the 2nd week showed signs of nerve fiber degeneration >95% by ENOG, while facial EMG provided information on the fibrillation potentials, and the characteristic of denervated muscles. Subsequently, similar outcome was observed after exposure to electrophysiological testing and clinical re-evaluation after 6 months rehabilitation, using the HB III-IV scale.

The hypoglossal facial nerve anastomosis remains the most popular technique applied in the reanimation of paralyzed faces [[26], [27], [28]]. The main goal of this surgery is to restore facial symmetry, during rest, and also while expressing emotions, demonstrating voluntary motions and ensuring the preserved tongue function [22,26,29]. Moreover, the end to end method was first proposed by Körte and Bernhard in 1901, where a neural graft was used to suture the hypoglossal to the facial nerve. This procedure was widely developed in the works of Balance and Duel in 1932 [30], and has been associated with some drawbacks, including palsy and hypotrophy of the ipsilateral tongue, leading to impaired swallowing and speech in 45% of patients [31]. However, Cusimano and Sekhar [27], and Arai et al. [23] developed a technique to prevent these functional defects. This required performing anastomosis between 30 and 50% of the ipsilateral hypoglossal nerve, to finely transect a split longitudinal and connect the facial nerve, projecting to the tongue, in order to prevent musculature atrophy.

Split hypoglossal facial anastomosis technique was used on both patients suffering SBF-mediated total facial paralysis on the right side for over 6 months duration. Furthermore, good recovery of facial reanimation with HB-1 scale was accomplished, and none experienced hemi tongue atrophy or dysfunction.

## 4 Conclusion

This report presented two cases of facial paralysis caused by SBF's, and treated with split hypoglossal facial anastomosis to restore functional reanimation. This outcome was adequately achieved after the procedure, and the hypoglossal nerve function was preserved.

## Provenance and peer review

Not commissioned, externally peer reviewed.

## Author contribution

Please specify the contribution of each author to the paper, e.g. study concept or design, data collection, data analysis or interpretation, writing the paper, others, who have contributed in other ways should be listed as contributors. EP, MCO, and MF researched the literature and wrote the manuscript. EP and MCO operated on the patient and had an idea for this case report. EP, MCO, and MF checked the manuscript and made corrections. EP, MCO and MF provided the overall guidance and support. All the authors read and approved the final manuscript.

## Declaration of competing interest

The authors declare that they have no conflict of interests.

## References

[1] Ghajar J. (2000). Traumatic brain injury. Lancet.

[2] Prasetyo E. (2020). The primary, secondary, and tertiary brain injury. Crit. Care Shock.

[3] Katzen J.T., Jarrahy R., Eby J.B., Mathiasen R.A., Margulies D.R., Shahinian H.K. (2003). Craniofacial and skull base trauma. J. Trauma.

[4] Varo Alonso M., Utrilla Contreras C., Díez Tascón Á., García Raya P.S., Martí de Gracia M. (2019). Traumatic injury of the petrous part of the temporal bone: keys for reporting a complex diagnosis. Radiologia.

[5] Feldman J.S., Farnoosh S., Kellman R.M., Tatum S.A. (2017). Skull base trauma: clinical considerations in evaluation and diagnosis and review of management techniques and surgical approaches. Semin. Plast. Surg..

[6] Chang C.Y., Cass S.P. (1999). Management of facial nerve injury due to temporal bone trauma. Am. J. Otol..

[7] Ringl H., Schernthaner R., Philipp M.O., Metz-Schimmerl S., Czerny C., Weber M., Gäbler C., Steiner-Ringl A., Peloschek P., Herold C.J., Schima W. (2009). Three-dimensional fracture visualisation of multidetector CT of the skull base in trauma patients: comparison of three reconstruction algorithms. Eur. Radiol..

[8] McELHANEY J.H., Hopper R.H., Nightingale R.W., Myers B.S. (1995). Mechanisms of basilar skull fracture. J. Neurotrauma.

[9] Agha R.A., Borrelli M.R., Farwana R., Koshy K., Fowler A.J., Orgill D.P., Zhu H., Alsawadi A., Noureldin A., Rao A., Enam A., Thoma A., Bashashati M., Vasudevan B., Beamish A., Challacombe B., De Wilde R.L., Machado-Aranda D., Laskin D., Muzumdar D., D’cruz A., Manning T., Healy D., Pagano D., Goel P., Ranganathan P., Pai P.S., Raja S., Ather M.H., Kadioäžlu H., Nixon I., Mukherjee I., Gómez Rivas J., Raveendran K., Derbyshire L., Valmasoni M., Chalkoo M., Raison N., Muensterer O., Bradley P., Roberto C., Afifi R., Rosin D., Klappenbach R., Wynn R., Giordano S., Basu S., Surani S., Suman P., Thorat M., Kasi V. (2018). The SCARE 2018 statement: updating consensus Surgical CAse REport (SCARE) guidelines. Int. J. Surg..

[10] Bello H.R., Graves J.A., Rohatgi S., Vakil M., McCarty J., Van Hemert R.L., Geppert S., Peterson R.B. (2019). Skull base–related lesions at routine head CT from the emergency department: pearls, pitfalls, and lessons learned. Radiographics.

[11] Kral T., Zentner J., Vieweg U., Solymosi L., Schramm J. (1997). Diagnosis and treatment of frontobasal skull fractures. Neurosurg. Rev..

[12] Brawley B.W., Kelly W.A. (1967). Treatment of basal skull fractures with and without cerebrospinal fluid fistulae. J. Neurosurg..

[13] Wani A.A., Ramzan A.U., Raina T., Malik N.K., Nizami F.A., Qayoom A., Singh G. (2013). Skull base fractures: an institutional experience with review of literature. Indian J. Neurotrauma..

[14] Faried A., Halim D., Widjaya I.A., Badri R.F., Sulaiman S.F., Arifin M.Z. (2019). Correlation between the skull base fracture and the incidence of intracranial hemorrhage in patients with traumatic brain injury. Chin. J. Traumatol..

[15] Mokolane N.S., Minne C., Dehnavi A. (2019). Prevalence and pattern of basal skull fracture in head injury patients in an academic hospital. SA J. Radiol..

[16] Brodie H.A., Thompson T.C. (1997). Management of complications from 820 temporal bone fractures. Am. J. Otol..

[17] Schuknecht B., Graetz K. (2005). Radiologic assessment of maxillofacial, mandibular, and skull base trauma. Eur. Radiol..

[18] Li J., Goldberg G., Munin M.C., Wagner A., Zafonte R. (2004). Post-traumatic bilateral facial palsy: a case report and literature review. Brain Inj..

[19] Felix H., Eby T.L., Fisch U. (1991). New aspects of facial nerve pathology in temporal bone fractures. Acta Otolaryngol..

[20] Reisser C., Schubert O., Forsting M., Sartor K. (1996). Anatomy of the temporal bone: detailed three-dimensional display based on image data from high-resolution helical CT: a preliminary report. Am. J. Otol..

[21] Sánchez-Ocando M., Gavilán J., Penarrocha J., González-Otero T., Moraleda S., Roda J.M., Lassaletta L. (2019). Facial nerve repair: the impact of technical variations on the final outcome. Eur. Arch. Oto-Rhino-Laryngol..

[22] Samii M., Alimohamadi M., Khouzani R.K., Rashid M.R., Gerganov V. (2015). Comparison of direct side-to-end and end-to-end hypoglossal-facial anastomosis for facial nerve repair. World Neurosurg..

[23] Arai H., Sato K., Yanai A. (1995). Hemihypoglossal-facial nerve anastomosis in treating unilateral facial palsy after acoustic neurinoma resection. J. Neurosurg..

[24] Kong K., Sevy A. (2017). Temporal bone fracture requiring facial nerve decompression or repair. Oper. Tech. Otolaryngol. Neck Surg..

[25] Xu P., Jin A., Dai B., Li R., Li Y. (2017). Surgical timing for facial paralysis after temporal bone trauma. Am. J. Otolaryngol..

[26] May M., Sobol S.M., Mester S.J. (1991). Hypoglossal-facial nerve interpositional-jump graft for facial reanimation without tongue atrophy. Otolaryngol. Neck Surg. Off. J. Am. Acad. Otolaryngol. Neck Surg..

[27] Cusimano M.D., Sekhar L. (1994). Partial hypoglossal to facial nerve anastomosis for reinnervation of the paralyzed face in patients with lower cranial nerve palsies: technical note. Neurosurgery.

[28] Pitty L.F., Tator C.H. (1992). Hypoglossal-facial nerve anastomosis for facial nerve palsy following surgery for cerebellopontine angle tumors. J. Neurosurg..

[29] Ozsoy U., Hizay A., Demirel B.M., Ozsoy O., Bilmen Sarikcioglu S., Turhan M., Sarikcioglu L. (2011). The hypoglossal–facial nerve repair as a method to improve recovery of motor function after facial nerve injury. Ann. Anat. - Anat. Anzeiger..

[30] Manni J.J., Beurskens C.H., van de Velde C., Stokroos R.J. (2001). Reanimation of the paralyzed face by indirect hypoglossal-facial nerve anastomosis. Am. J. Surg..

[31] Hammerschlag P.E. (1999). Facial reanimation with jump interpositional graft hypoglossal facial anastomosis and hypoglossal facial anastomosis: evolution in management of facial paralysis. Laryngoscope.

